# The Impact of Surface Treatment and Degree of Vacuum on the Interface and Mechanical Properties of Stainless Steel Clad Plate

**DOI:** 10.3390/ma11091489

**Published:** 2018-08-21

**Authors:** Ying-ying Feng, Huan Yu, Zong-an Luo, Guang-ming Xie, R. D. K. Misra

**Affiliations:** 1 The State Key Laboratory of Rolling and Automation, Northeastern University, Shenyang 110819, China; huinanmu@126.com (H.Y.); huinanmu@163.com (Z.-a.L.); huinanmu-2001@163.com (G.-m.X.); 2 Department of Metallurgical, Materials and Biomedical Engineering, University of Texas, El Paso, TX 79968, USA; fengyingying-1982@163.com

**Keywords:** clad plate, vacuum hot rolling, surface treatment, degree of vacuum, interface inclusions

## Abstract

In this study, the impact of different surface treatment and degree of vacuum on the interface and mechanical properties of 304/Q345 stainless steel clad plate was investigated. The study indicated that more continuous or aggregated Al_2_O_3_ and Si-Mn composite oxides were formed at the interface after brush grinding. However, less inclusions such as Al_2_O_3_, MnS and Ca-Mg-Al-Si composite oxides were formed at the interface after pickling treatment. For the vacuum degrees of 10^−2^ Pa, 1 Pa and 105 Pa, the oxidation reaction became more intense with the decrease in vacuum degree. The interface inclusions were gradually changed from Al_2_O_3_ and Si-Mn complex oxides to oxide scale and MnCr_2_O_4_ spinel oxide. The interfacial bonding strength of stainless steel clad plate was improved with the increase in degree of vacuum. The bonding strength was 55 MPa at vacuum of 10^5^ Pa, but it was 484 MPa at vacuum of 10^−2^ Pa, which is far greater than that of the national standard, and an excellent performance was obtained.

## 1. Introduction

The stainless steel clad plate is a new means of processing dissimilar materials. The clad plate consists of thicker carbon steel as a substrate and thinner stainless steel as a cladding material. Stainless steel clad plates combine the excellent corrosion resistance of stainless steel and the good mechanical properties and low cost of carbon steel. This has led to the stainless steel clad plates being widely used in the chemical, petroleum, and shipbuilding industry, etc. [1,2].

At present, there are many methods for producing stainless steel clad plate, among which, hot roll-cladding is the most economical and efficient manufacturing process [3,4]. However, there are some shortcomings in the preparation process of hot roll-cladding plates, such as easy oxidation of the bonding interface, which results in remarkably reduced interface bonding strength. Therefore, integral and continuous bonding between the cladding metal and the base metal must be provided to obtain good shear strength of the bonded interface through an appropriate surface treatment for the hot roll-cladding method [5,6]. Additionally, the final properties of clad plates at the heating stage are significantly affected by the high-temperature oxidation products of the interface.

The usual method to solve the problem of bonding interface oxidation is to isolate the outside atmosphere during the preparation process by, for example, welding around the base cladding plates and vacuum extraction [7], vacuum electron beam welding (VEBW) [8,9] and other methods. Quadir et al. [10] found that a bond was not easily achieved in aluminum alloys after heating to elevated temperatures of 573–823 K in air due to the increase of surface oxidation. Li et al. [11] studied the amount of oxides at the interface of stainless steel clad plates at different degrees of vacuum, and the results showed that the surface oxidation was more obvious at lower vacuum. Wei et al. [12] used manual arc welding and vacuum pumping to vacuum blanks to study the process of extremely thick clad plates. They showed that under a certain rolling process, there were large oxides at the interface and no compound area existed in the head and tail of the clad plate. Zu et al. [13] reduced the interfacial oxidation by rapid induction heating and applying protective gas, and the interfacial bonding strength was 316 MPa. The study reported that hot-rolled composite interface oxides were caused by oxidation of alloying elements with oxygen at high temperatures. However, there are few investigations on the influence of different surface treatment methods and different degree of vacuum on the mechanism of formation of oxides and the mechanical properties of the interface for stainless steel clad plates.

In this study, stainless steel clad plates were prepared by Vacuum Rolling Cladding (VRC) technology, which ensured that the plates had extremely excellent interface bonding performance with rare oxidation due to the use of vacuum electron beam welding (VEBW) technology. We studied the influence of two different surface treatment methods, brush grinding and pickling treatment, and three different vacuum degrees, which were 10^−2^ Pa, 1 Pa and 10^5^ Pa. The formation mechanism of oxides and the influence on mechanical properties are analyzed, thus providing a reference for controlling the oxidation products of hot-rolled stainless steel clad plates.

## 2. Materials and Experimental Procedures

The base and clad materials of the clad plates were Q345 carbon steel and 304 austenitic stainless steel, respectively, and the chemical composition of both these materials are shown in Table 1. The dimensions of the stainless steel and Q345 were 150 mm × 100 mm × 6 mm and 150 mm × 100 mm × 60 mm, respectively.

**Figure 1:**
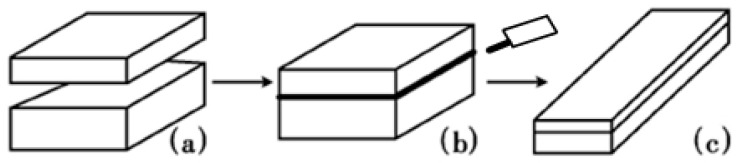
Schematic of vacuum rolling cladding. (**a**) Surface cleaning and blanks assembling; (**b**) vacuum electron beam welding (VEBW); (**c**) Hot rolling.

A schematic of vacuum roll-cladding (VRC) is shown in Figure 1, including four steps, surface cleaning, assembly, VEBW, heating and rolling. The detailed process is: the surface of the blanks is cleaned first, and after assembly, the blanks are welded along four sides of the contact surface of the two billets in a high vacuum environment using an electron beam welding machine, then, the clad plate are used for high-temperature oxidation experiments and hot rolling experiments.

The nature, composition, morphology and formation mechanism of oxide inclusions at composite interface, as well as the impact on mechanical properties for different methods of surface treatment and different degrees of vacuum are discussed based on the high temperature oxidation experiments and hot rolling experiments.

First, the surface of the billets was cleaned by means of brush grinding and pickling, respectively, to remove contaminants and the oxide layer. Two sets of billets were prepared in each method. The billets were then electron beam welded at vacuum of 10^−2^ Pa. One set was used for high temperature oxidation experiments and the other set was used for hot rolling experiments. Similarly, to study the effect of vacuum on the oxidation products, two sets of billets were combined by pickling, and the stacked billets were welded under vacuum of 1 Pa and 10^5^ Pa for high temperature oxidation and hot rolling experiments (the billets at 10^−2^ Pa high vacuum were prepared as described above). The above-obtained stacked billets were placed in an electric resistance furnace and heated to 1200 °C for 2 h. The billets for the high-temperature oxidation test were directly cooled to room temperature after heating while the hot rolling experiment were carried out on a 450 Two-High Hot Rolling Mill made by NEU. The rolling speed was 1.0 m/s, and the total reduction rate was 50%.

The specimens for microstructural examination were obtained from the central part of the clad plates in a plane parallel to the rolling direction, and the specimen was etched with 4% nitric acid alcohol solution when picking was adopted as the surface treatment. The interfacial microstructure was observed using optical microscope (OM, Leica, Wetzlar, Germany), scanning electron microscopy (SEM, Oberkochen, Germany), energy dispersive spectroscopy (EDS, Oxford, Abingdon, UK) and transmission electron microscope (TEM, Hillsboro, OR, USA). The composition and type of interfacial inclusions were determined by electron probe microanalyses (EPMA, JEOL, Tokyo, Japan) in conjunction with wavelength dispersive spectroscopy (WDS, JEOL, Tokyo, Japan).

**Figure 2:**
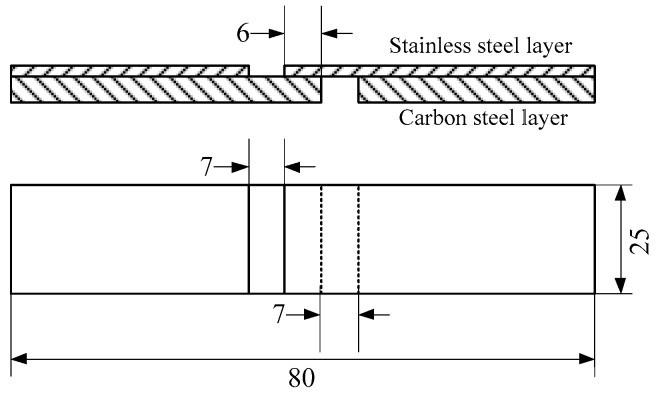
Schematic of shear test specimens (in mm).

For clad plates, the bond strength of the composite interface is the most important performance, and the shear strength of the interface bond strength will determine the main properties of the clad plates. In this paper, the bonding strength of the interface of stainless steel clad plate was tested by shear testing. According to the standard of GB/T 6396-2008 [14], the clad plates after hot rolling was cut into standard shear samples by wire cutting, and the dimensions are shown in Figure 2. The test was carried out on an electronic universal testing machine.

## 3. Results and Discussion

### 3.1. Influence of Surface Treatment Method on Interface Inclusions

**Figure 3:**
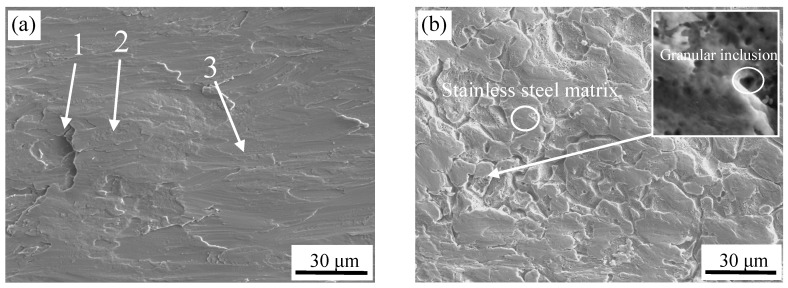
Stainless steel surface micrographs by different surface treatment. (**a**) brush grinding, and (**b**) pickling.

The SEM images of the interface for different surface treatments are shown in Figure 3. It can be seen that the surface of the stainless steel after brush grinding was not flat and exhibited an irregular shape. The stainless steel contains a high content of Cr, which can form a stable Cr_2_O_3_ passive film at atmosphere or in the presence of oxidizing medium. During the process of brush grinding, the Cr_2_O_3_ passive film on the surface of the stainless steel was lifted by the grinding force, and was subsequently covered irregularly on the surface of stainless steel. Three different positions for chemical composition analysis were used, as shown in Figure 3a. The WDS analysis of these three positions on the stainless steel surface is shown in Table 2. O element was observed at the third point. Thus, region 3 was stainless steel substrates. O was present at the first and second point, and the content of oxygen in region 1 was greater than region 2. The content of O decreases with increasing depth based on the structural characteristics of stainless steel passive film [15], which indicated that region 1 was the outer layer of the passive film on the surface of stainless steel, and region 2 was the secondary outer layer. Also, the surface at region 1 was not polished for its lower position.

Figure 3b shows a valley-like surface after pickling, and it can be seen that more granular inclusions were formed at the bottom of valley, as shown in the higher magnification image. Valleys are formed at the dislocations, inclusions, and other defects at the grain boundaries in the steel matrix, and can be easily corroded by the acid solution [16,17].

**Figure 4:**
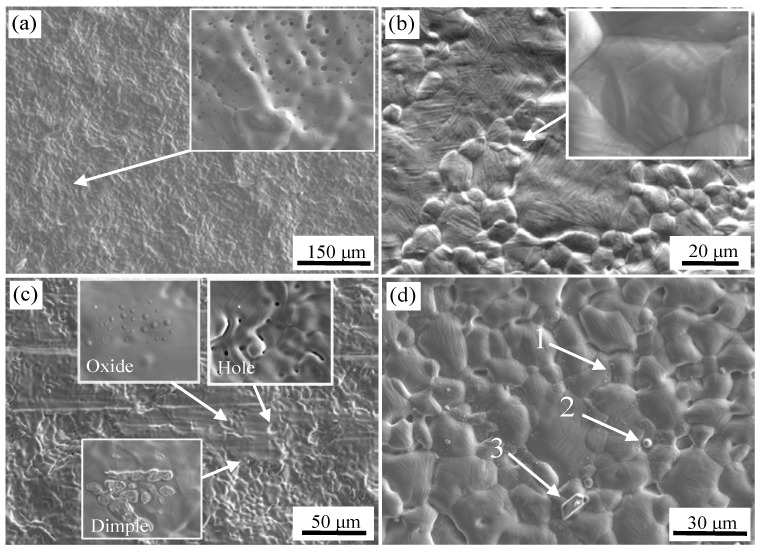
Surface morphology of stainless steel and carbon steel under different surface treatments after high temperature oxidation experiment; After brush grinding (**a**) stainless steel side; (**b**) carbon steel side and after pickling (**c**) stainless steel side; (**d**) carbon steel side.

The SEM images of the surface of 304 stainless steel and carbon steel after high-temperature oxidation experiments for the two kinds of surface treatment are shown in Figure 4. It can be seen that the irregular lamellar shape of the stainless steel surface after brush grinding was basically eliminated in Figure 4a, but the surface was still not flat and initial joining did not appear on the composite surface. The stainless steel substrate was austenite after cooling. With the growth of grains, the surface of the stainless steel exhibited some ups and downs, but not obviously. Moreover, the physicochemical properties of the two metals were quite different, and they required more pressure to generate bonding. The clamping force was insufficient to form the initial bonding. The inclusions were not detected in the flat area of stainless steel in the enlarged area by WDS analysis. A number of circular inclusions of different sizes were formed in depressions [18]. The main constituents were O: 48.0 at %, Al: 5.2 at %, Si: 10.0 at %, Mn: 4.3 at %, Cr: 4.2 at %, Ni: 0.9 at %, and the balance was Fe.

**Figure 5:**
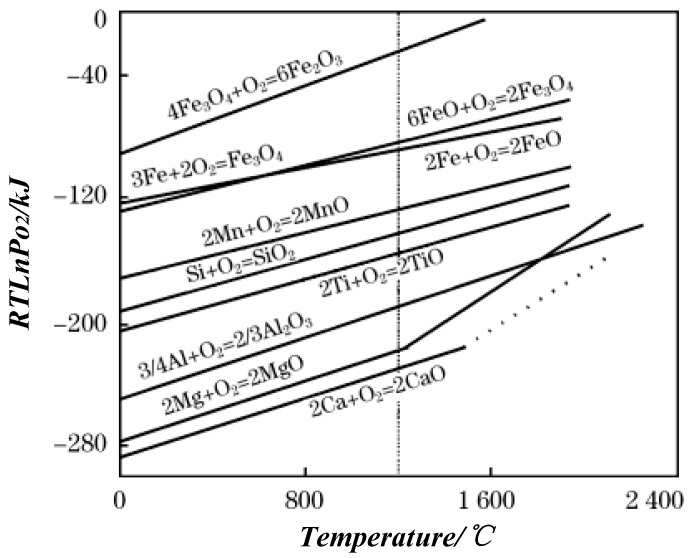
ΔG_0_–T relationship of metal oxidation reaction.

When the metal is oxidized, the free energy (ΔG_0_) depending on metal oxide is formed, which indicates the affinity between the metal and oxygen. The lower the value, the higher the stability of oxide and easier the metal is oxidized under lower free energy [19]. Figure 5 shows the ΔG_0_–T diagram of the metal oxidation reaction [20], which is used to determine the possibility of an oxidation reaction. When the temperature is 1200 °C, 304 stainless steel and Q345 carbon steel are expected to form complex oxidation. The ΔG_0_ of Al, Si, Mn, Cr, and Fe oxides are different, and the increasing sequence is ΔG_Al2O3_ < ΔG_SiO2_ < ΔG_MnO_ < ΔG_Cr2O3_ < ΔG_FeO_ < ΔG_Fe3O4_ < ΔG_Fe2O3_, that is, the affinities of Al, Si, Mn, Cr, Fe, with O decrease. Thus, Al was preferentially oxidized to Al_2_O_3_. Meanwhile, Al diffused to the surface due to concentration difference. The content of oxygen in the valley or depressed surface region is high, and Al below the surface did not diffuse to the interface to react. While Al on the surface was consumed, the oxygen continued to react with Si and Mn. However, the oxygen content was limited, which resulted in the formation of composite inclusions dominated by Al_2_O_3_ and Si-Mn oxides.

Meanwhile, the protruded grain and the depressed grain boundary of the carbon steel can be seen in Figure 4b. This is because, in the cooling process, the process of grain growth and recrystallization was completed based on sufficient phase transition in carbon steel. When the undercooled austenite in carbon steel changes to ferrite, pearlite and martensite, the volume expansion occurs. However, the expansion is small because of the presence of carbides at the grain boundaries. Martensite was also observed within grains of carbon steel [18,21,22], as shown in Figure 4b, which indicated that the microstructure of carbon steel near the interface was transformed to martensite on cooling without rolling.

**Figure 6:**
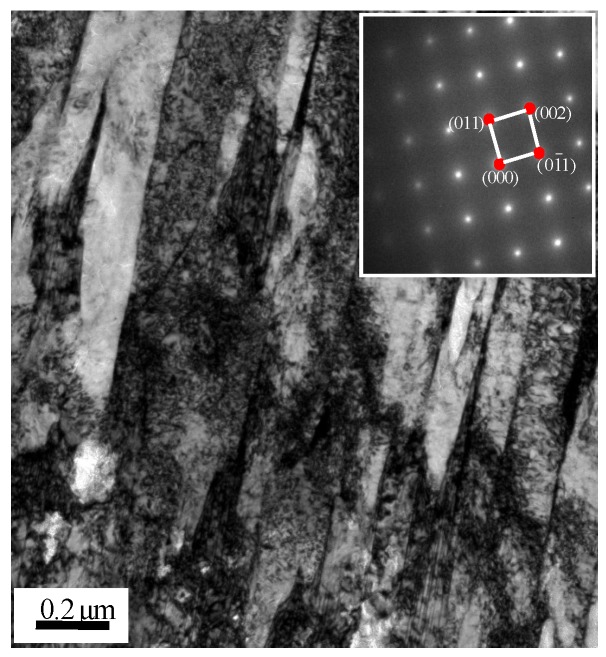
TEM micrograph and diffraction pattern of martensite.

In order to further verify the presence of martensite, TEM specimens were prepared by an ion thinning method. The microstructure of the composite region was clearly observed. A large number of lath martensite structures and diffraction spots of bcc martensite were observed in Figure 6, which confirmed the existence of martensite. The chemical composition of carbon steel surface inclusions was studied by WDS and was O: 20.5 at %, Al: 10.3 at %, Si: 0.6 at %, Mn: 2.2 at %, Cr: 7.1 at %, Ni: 0.7 at %, and the balance was Fe, and Al_2_O_3_ and Si-Mn oxides were formed according to selective oxidation.

**Figure 7:**
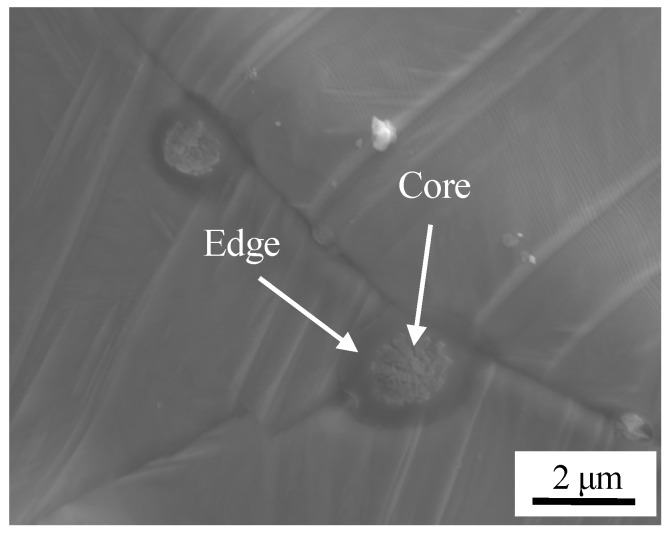
Morphology of oxide.

The surface image of stainless steel after pickling is shown in Figure 4c. There were three features; holes, oxide inclusions, and a small number of dimples. Residual sulcus after pickling were not eliminated during high temperature oxidation, and circular holes were formed. A certain degree of metallurgical bonding formed in the localized area of the surface during high temperature oxidation stage. An initial bond formed, and dimples were formed after separation at the initial bond point. A certain number of small oxides were formed at the grain boundaries, and the enlarged view is shown in Figure 7. The chemical composition was detected by WDS, O: 24.8 at %, Mg: 16.5 at %, Al: 6.4 at %, Si: 1.5 at %, Ca: 8.1 at %, Mn: 3.8 at %, the balance was Fe at the core of oxide. O: 20.7 at %, Mg: 1.8 at %, Al: 3.2 at %, Si: 3.6 at %, Ca: 0.6 at %, Mn: 3.8 at %, the balance Fe was detected on the shell of oxide. So, the composite oxides mainly consisted of a core of Ca, Mg, and Al oxides and a shell of Si and Mn oxides.

Considering that the stainless steel billet experienced deoxidation during the smelting process, oxides and silicates were formed. At the same time, a little amount of oxygen, sulfur, aluminum and other impurity elements remained in the steel in the form of compounds or elements [23], and the complex oxide inclusions such as SiO_2_, Al_2_O_3_, MgO and CaO were formed in stainless steel. After pickling, oxygen accumulated in the valley of the grain boundary, and the concentration was high. During heating, oxygen preferentially reacts with Ca, Mg and Al due to selective oxidation. Then, Si and Mn reacted with oxygen to form oxides, and complex oxides were eventually formed.

The surface of the carbon steel after pickling is shown in Figure 4d, which also shows the morphology of the protruded grain and the depressed grain boundary, and the phase transformation of martensite as described above. Three characteristic locations were selected for component analysis, as identified in Figure 4d. The chemical composition of the carbon steel surface was detected by WDS, N: 27.1 at %, Ti: 5.8 at %, Si: 1.0 at %, Mn: 1.2 at %, with the balance Fe at point 1. At the second point, S: 49.9 at %, Mn: 44.8 at %, with the balance Fe. At the third point, only Al and O were detected. The inclusions at the grain boundary were TiN, granular MnS, and Al_2_O_3_. At the temperature of 1200 °C, the standard free energy of TiN is the lowest among all metal nitrides. Thus, TiN were formed. A large number of small TiN inclusions were formed at the grain boundaries. During smelting, a certain amount of Mn was added to the steel in order to avoid the formation of a hot brittle phase of FeS. Thus, Mn and S combined at high temperature to form MnS, and was stable even at 1200 °C. Al_2_O_3_ was formed based on the rule of selective oxidation as described above.

**Figure 8:**
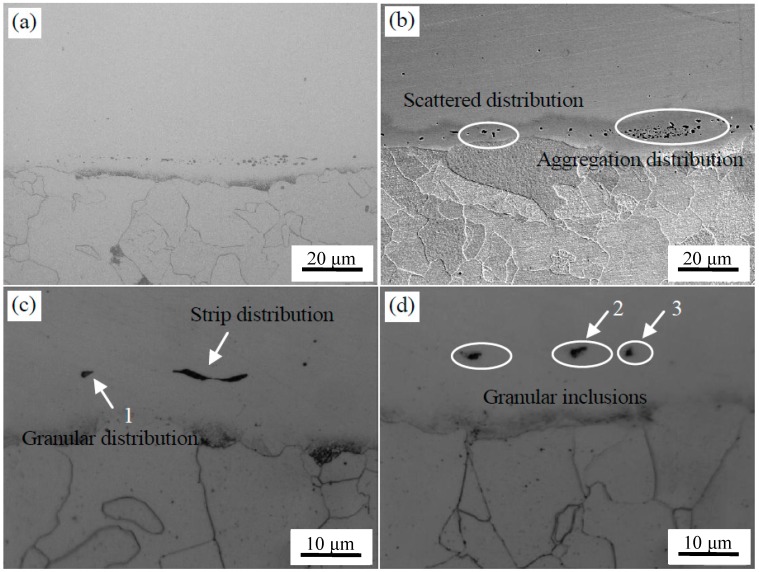
Microstructure of stainless steel clad plate with two treatments after hot rolling experiment. After brush grinding: (**a**) metallographic photo and (**b**) SEM. After pickling treatment: (**c**) metallographic photo and (**d**) SEM.

The OM images of the composite interface of clad plates with two surface treatments after the hot rolling are shown in Figure 8. It can be seen that the bonding interface was relatively straight, and excellent bonding was achieved without any unwelded zones after rolling.

**Figure 9:**
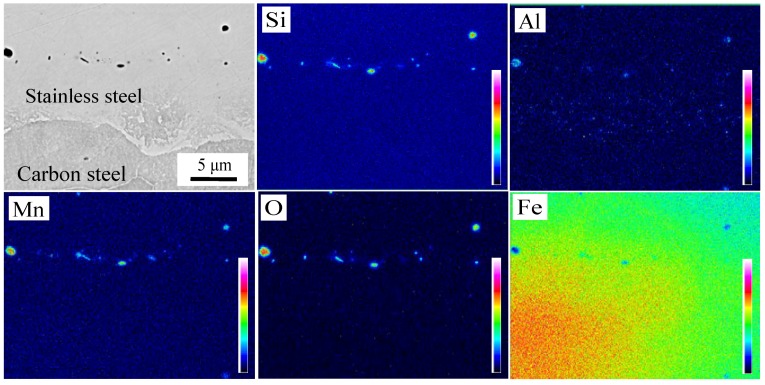
Distribution of elements in oxide after brush grinding.

The microstructure of the interface after brush grinding is presented in Figure 8a,b. There were two kinds of inclusions with different morphologies at the composite interface. That is, the sporadic distribution of particles and the short rod-like segregation into the interfacial distribution along the band. Nomura et al. [24] found that Si-Mn oxides were easily formed on the surface of steel containing Si and Mn, which are considered to be sensitive to oxidation. The machined carbon steel and stainless steel surface adsorbed O on the surface. These O atoms at the interface reacted preferentially with the easily oxidized elements, which were Si and Mn on both sides of the interface during prolonged heating and formed a thin layer of Si-Mn oxides. The oxidation layer was dispersed at the composite interface after rolling. The EPMA analysis of the composite interface is shown in Figure 9. Elements Al, Si, Mn, and O were segregated near the interface. The segregation of Al element is less, and Al_2_O_3_ inclusions were preferentially generated based on the principle of selective oxidation. However, Si and Mn had a higher amount of segregation and reacted with the remaining O; Si-Mn oxides were finally formed.

**Figure 10:**
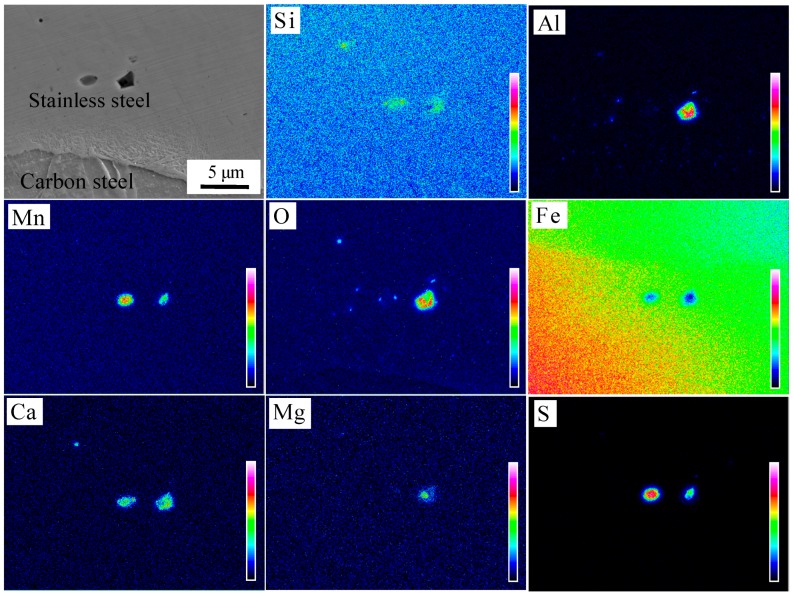
Distribution of elements in oxide after pickling.

The microstructure of the interface after pickling is shown in Figure 8c,d. It can be seen that fewer interface inclusions were present, mainly in the form of granules or strips near the interface compared with the brush grinding. Three typical locations were selected for component analysis, as identified in Figure 8c,d. The chemical composition of inclusions at the interface was detected by WDS, O: 18.8 at %, Al: 10.4 at %, Si: 0.7 at %, Cr: 8.2 at %, Mn: 1.5 at %, Ni: 1.6 at %, with the balance Fe at point 1; O: 21.2 at %, Al: 1.8 at %, Si: 4.1 at %, S: 17.8 at %, Ca: 1.9 at %, Cr: 7.6 at %, Mn: 18.8 at %, Ni: 2.2 at %, with the balance Fe at point 2; and O: 31.2 at %, Al: 6.0 at %, Si: 7.2 at %, Ca: 3.5 at %, Mg: 1.8 at %, Cr: 6.6 at %, Mn: 3.5 at %, Ni: 0.8 at %, with the balance Fe at point 3. The main types of inclusions were Al_2_O_3_, MnS, and Ca-Mg-Al-Si mixed oxide. The distribution and types of inclusions at the interface were analyzed by EPMA, as seen in Figure 10. The mechanism of formation of MnS and Al_2_O_3_ inclusions has been described before, but the size decreased and the distribution became more diffuse under the effect of larger rolling force. The Ca-Mg-Al-Si composite oxide was derived from the small round oxide, when the 304 stainless steel was oxidized at high temperature. The types of inclusions were consistent with those formed in high temperature oxidation experiments, which indicated that the interfacial inclusions after hot rolling were all formed during the heating stage.

In general, continuous or aggregated Al_2_O_3_ and Si-Mn composite oxides formed at the composite interface after brush grinding. A small amount of oxides such as Al_2_O_3_, MnS, etc. were generated at the composite interface after pickling. The oxide inclusions after hot rolling were formed during the high-temperature oxidation stage. Therefore, the interface inclusions after pickling were small and less, and interface oxidation was greatly reduced.

### 3.2. Impact of Vacuum Degree on Interface Inclusions

Through the study for interface inclusions under different surface treatment methods, it was found that the interface inclusions of clad plates were less after pickling. The surface treatment by pickling was used to study the formation of oxidations at the composite interface at high temperature with different degrees of vacuum.

**Figure 11:**
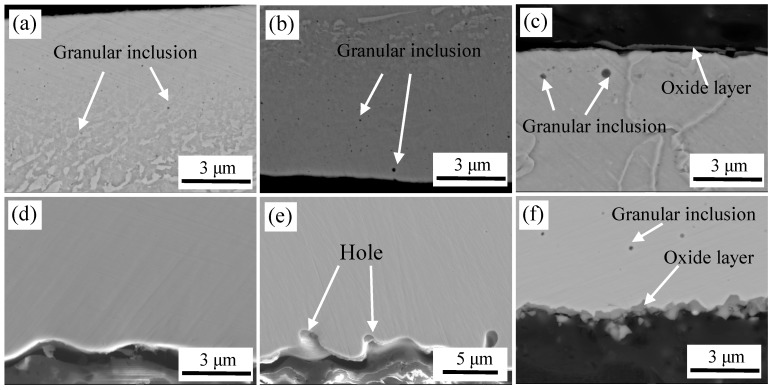
The cross-sectional images of high temperature oxidation at different degrees of vacuum. At 10^–2^ Pa: (**a**) carbon steel and (**d**) stainless steel; at 1 Pa: (**b**) carbon steel and (**e**) stainless steel; and at 10^5^ Pa: (**c**) carbon steel and (**f**) stainless steel.

Figure 11 shows the microstructure of a cross section of interface after high-temperature oxidation at different degrees of vacuum. Figure 11a,d are micrographs at vacuum of 10^−2^ Pa. It can be seen that the surface layer of carbon steel was oxidized, and many fine granular inclusions were dispersed on the surface. It was only enriched with Al and O based on WDS analysis (except matrix elements), and the atomic ratio of Al to O was 2:3. Thus, the inclusions were Al_2_O_3_. The cross section of stainless steel was clean and clear without oxide. Figure 11b,e are images at a vacuum level of 1 Pa. Carbon steel surface layers also undergo oxidation. As the vacuum level decreased, the content of oxygen increased, and the number, density and the size of oxide inclusions were all increased. Al_2_O_3_ and Si-Mn composite oxides were detected.

The cross section of stainless steel was relatively clean, but there were a certain number of holes in the localized area, which were formed on the stainless steel surface after pickling and could not be completely eliminated during heating at a high temperature. Thus, a large amount of oxygen is trapped here, which easily reacted with the active metals in the stainless steel to form oxides. Figure 11c,f are images at vacuum of 10^5^ Pa. With the decrease in vacuum, the oxide content clearly increased, and an oxide layer and larger oxides were formed on the carbon steel side near the interface. At high-temperature and high-oxygen content, Fe was oxidized rapidly to form an oxide layer. Because sufficient oxygen was enriched on the surface, Al_2_O_3_ and Si-Mn composite oxides were sequentially generated based on the preferential selective oxidation mechanism. Figure 11f shows that oxides were also formed on the side of the stainless steel, which contained high content of Si, Mn, Cr, Fe according to WDS analysis, and eventually the Si-Mn-Cr-Fe composite oxides were formed. When oxidation occurred, Si-Mn composite oxide is first formed based on the principle of selective oxidation, when elements of Si and Mn are depleted, the remaining O reacts with Cr and Fe.

**Figure 12:**
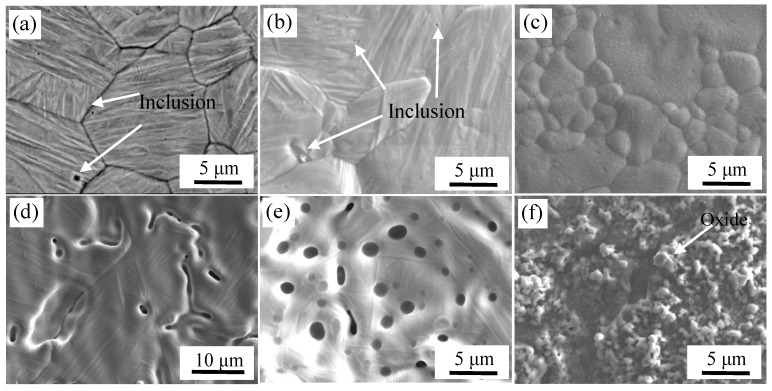
High temperature oxidation images of steel surface at different vacuum degree. At 10^–2^ Pa: (**a**) carbon steel and (**d**) stainless steel; at 1 Pa: (**b**) carbon steel and (**e**) stainless steel; and at 10^5^ Pa: (**c**) carbon steel and (**f**) stainless steel.

The microstructure of high-temperature oxidation of stainless steel and carbon steel at different vacuum degree are shown in Figure 12.

Micrographs of carbon steel and stainless steel corresponding to the vacuum of 10^−2^ Pa are shown in Figure 12a,d. The surface inclusions of carbon steel were mainly TiN, MnS and Al_2_O_3_ after pickling in the high-temperature oxidation experiments. A small amount of Ca-Mg-Al-Si composite oxides were formed on the surface of stainless steel according to high temperature oxidation experiments. The forming mechanism of surface inclusions has been discussed in Section 3.1.

Morphologies of the samples with a vacuum of 1 Pa are shown in Figure 12b,e. It can be seen that the surface of carbon steel was similar to that in the high vacuum, there was no large-scale oxidation, and only a small amount of oxide was formed. O: 21.5 at %, Al: 4.7 at %, Si: 5.6 at %, Mn: 4.6 at % and the balance of Fe were detected by WDS. Al_2_O_3_ and Si-Mn composite oxides were detected and distributed on the surface because of selective oxidation. With the decrease in vacuum, the content of oxides was increased on the surface of the depressions for stainless steel, and larger Al-Si-Mn oxides were formed. When the vacuum was 1 Pa, the interface was in a low-vacuum sealed state. Although the content of oxygen at the lower vacuum was higher than that under the higher vacuum, and the initial oxygen partial pressure was also high, the total oxygen content was limited. With the oxidation reaction, oxygen was gradually consumed until the partial pressure of oxygen was reduced to a level such that oxide was not formed. Thus, the composite interface did not undergo severe oxidation at lower vacuum, but was similar to the degree of reaction at high vacuum and a had a slightly higher degree of oxidation. The result was an increase in oxide production compared to the high vacuum [25].

**Figure 13:**
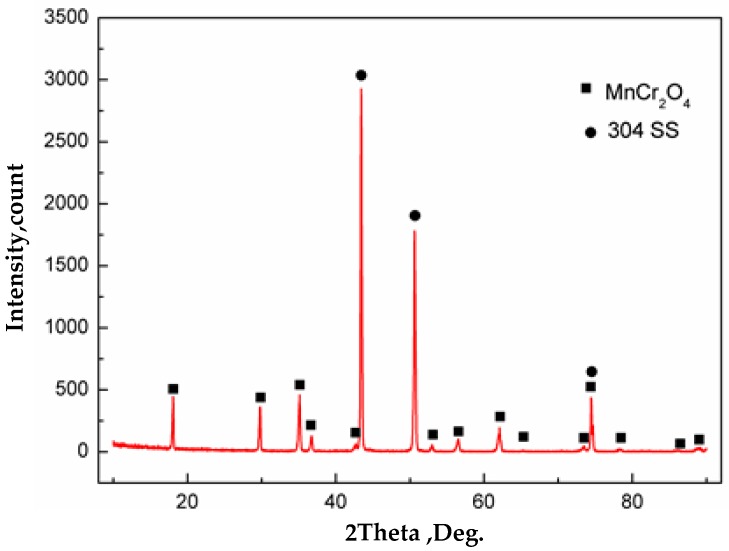
Phase analysis by X-ray diffraction of stainless steel surface after heating in vacuum of 10^5^ Pa.

Morphologies of samples with the vacuum of 10^5^ Pa are shown in Figure 12c,f. It can be seen that the surface of carbon steel and stainless steel were completely covered with oxides. The chemical composition of carbon steel surface was detected by WDS, which were O: 37.3 at %, Si: 0.8 at %, and the balance was Fe. Thus, oxides were formed on the surface of carbon steel. Severe oxidation of iron and oxygen occurred at 1200 °C. The chemical composition of stainless steel surface was O: 51.5 at %, Cr: 24.0 at %, Mn: 12.6 at % and the balance of Fe, as detected by WDS. Also, based on the XRD analysis of the surface of stainless steel as shown in Figure 13, almost all the inclusions on the surface of stainless steel were MnCr_2_O_4_. The composite interface was in contact with the outside atmosphere at vacuum of 10^5^ Pa. During subsequent heating, the stainless steel was severely oxidized. Due to the high content of Cr in stainless steel, a layer of Cr_2_O_3_ protective film was first formed on the surface of stainless steel at high-temperature and high-oxygen conditions according to the selective oxidation mechanism. With the progression of the oxidation, Cr_2_O_3_ was continuously generated and covered the entire stainless steel surface, which isolated the stainless steel matrix from the atmosphere. If Cr continues to oxidize on the composite surface, alloying elements must diffuse through the Cr_2_O_3_ layer to the surface and react with oxygen. Meanwhile, the lattice diffusion coefficient of Mn ions in Cr_2_O_3_ was two orders of magnitude higher than that of Cr ions, and its diffusion rate was faster than Si, Fe and Ni in stainless steel [26]. Therefore, Mn in the stainless steel matrix is the first to diffuse to the surface of Cr_2_O_3_ and reacts with oxygen to form MnO. When Cr_2_O_3_ and MnO are present on the surface of stainless steel, MnCr_2_O_4_ spinel oxides are formed [27,28].

Thus, when the vacuum was reduced, the degree of interfacial oxidation became more serious, which was unfavorable to obtaining the excellent interface of the hot-rolled clad plate. Therefore, the degree of vacuum at the composite interface should be controlled between 1 Pa and10^−2^ Pa, or less.

### 3.3. Mechanical Properties of Stainless Steel clad Plates at Different Vacuum 

**Figure 14:**
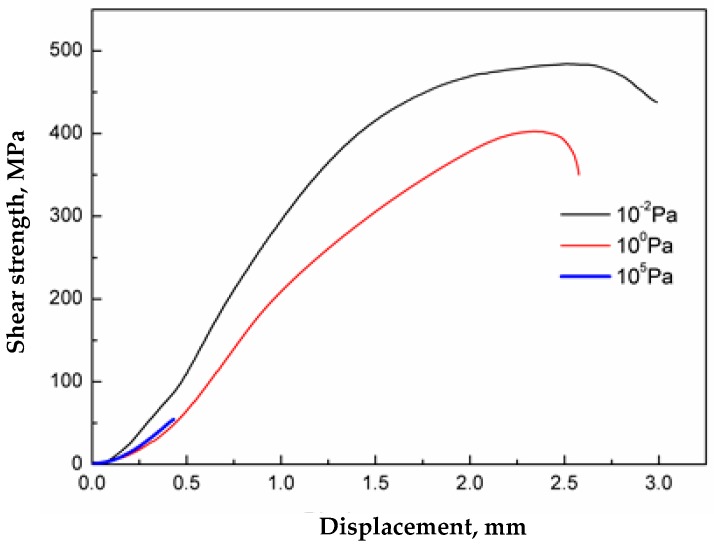
Shear strength-displacement curves of stainless clad steel interface at different vacuum degree.

The shear strength-displacement plot of the interface of stainless steel clad plate at different degrees of vacuum after pickling is shown in Figure 14.

Thus, with a decrease in the degree of vacuum, the bond strength of the composite interface became less. The interfacial shear strength of stainless steel clad plates prepared at high vacuum, low vacuum and atmospheric pressure was 484 MPa, 397 MPa and 55 MPa, respectively. In addition, with the decrease in vacuum degree, the deformation of the specimen in the shear test became smaller, which indicated that the deformation resistance of the composite interface also deteriorated when the degree of vacuum decreases. In summary, inclusions at the composite interface at different vacuum degrees was the key factor affecting the strength and toughness of the composite interface. Therefore, welding pickled stainless steel clad plates in high vacuum and hot rolling can provide excellent bonding performance.

## 4. Conclusions

1. At high temperature, the oxides that formed at the interface after brush grinding were continuous or aggregated Al_2_O_3_, Si-Mn composite oxides. A small amount of dispersed Al_2_O_3_, MnS and Ca-Mg-Al-Si composite oxides were formed after pickling, indicating that a cleaner interface can be obtained by pickling. Additionally, the type of oxide inclusions at the interface after hot rolling was unchanged, the inclusions were formed at the stage of high-temperature oxidation.
2. The lower the vacuum level, the more intense the oxidation of the composite interface. When the vacuum was 1 Pa and 10^−2^ Pa, the content of oxide inclusions was very small, and were mainly Al_2_O_3_ and Si-Mn composite oxides. When the vacuum was 10^5^ Pa, the composite interface was almost completely covered with an iron oxide skin and MnCr_2_O_4_ spinel oxides. Thus, during vacuum hot rolling cladding to prepare stainless steel clad plate, the vacuum at the composite interface should be controlled between 1 Pa–10^−2^ Pa, or less.
3. The interfacial shear strength of stainless steel clad plate after pickling at different vacuum degrees decreased with the decrease in vacuum degree, and was 484 MPa at 10^−2^ Pa, 397 MPa at 1 Pa and 55 MPa at 10^5^ Pa. The mechanical property of the stainless steel clad plate was mainly affected by the type and morphology of interface inclusions.

## Figures and Tables

**Table 1:** Chemical composition of clad plate materials (mass fraction %).

Element	C	Si	Mn	P	S	Al	Cr	Ni	Nb + V + Ti	Fe
Q345	0.2	0.55	1.6	0.035	0.035	0.015	-	-	Trace	Bal.
304	0.05	1.00	2.00	0.022	0.008	0.005	19.1	8.5	-	Bal.

**Table 2:** Wavelength dispersive spectroscopy (WDS) analysis of the three points identified in Figure 3a.

Position	1	2	3
O/at %	11.8	5.9	-
Si/at %	1.0	1.0	0.9
Cr/at %	16.2	17.3	20.0
Mn/at %	1.4	1.5	1.8
Fe/at %	64.0	68.0	70.9
Ni/at %	5.6	6.3	6.5

## References

[1] Yang M.N., Zuo X.Q., Zhao M.W., Wang J. (2012). Research Progress of Manufacturing Technology for Stainless Steel Clad Plate. Hot Work Tech..

[2] Luo Z.A., Wang G.L., Xie G.M., Wang L.P., Zhao K. (2013). Interfacial Microstructure and Properties of a Vacuum Hot Roll-bonded Titanium-Stainless Steel Clad Plate with a Niobium Interlayer. Acta Metall. Sin..

[3] Jing Y.A., Qin Y., Zang X., Sheng Q., Song H. (2014). A Novel Reduction-Bonding Process to Fabricate Stainless Steel Clad Plate. J. Alloys Compd..

[4] Jing Y., Qin Y., Zang X., Li Y. (2014). The Bonding Properties and Interfacial Morphologies of Clad Plate Prepared by Multiple Passes Hot Rolling in a Protective Atmosphere. J. Mater. Process. Technol..

[5] Zhu Z., He Y., Zhang X., Liu H. (2016). Effect of Interface Oxides on Shear Properties of Hot-Rolled Stainless Steel Clad Plate. Mater. Sci. Eng. A.

[6] Li L., Yin F.X., Nagai K. (2011). Progress of Laminated Materials and Clad Steels Production. Mater. Sci. Forum.

[7] Jing C., Tong J., Ren X. (2007). Bonding Behavior of 25Cr5MoA/Q235 Hot Rolled Clad Plates. J. Univ. Sci. Technol. B.

[8] Yan J.C., Zhao D.S., Wang C.W., Wang L.Y., Wang Y., Yang S.Q. (2013). Vacuum Hot Roll Bonding of Titanium Alloy and Stainless Steel Using Nickel Interlayer. Met. Sci. J..

[9] Zhao D.S., Yan J.C., Wang Y. (2009). Relative Slipping of Interface of Titanium Alloy to Stainless Steel during Vacuum Hot Roll Bonding. Mater. Sci. Eng. A.

[10] Quadir M.Z., Wolz A., Hoffman M. (2008). Influence of processing parameters on the bond toughness of roll-bonded aluminium strip. Scr. Mater..

[11] Li L., Zhang X.J., Liu H.Y. (2013). Formation Mechanism of Oxide Inclusion on the Interface of Hot-Rolled Stainless Steel Clad Plates. J. Iron Steel Res..

[12] Wei Y., Zhang Y.M., He C.Y. (2011). Rolling Compound Production of Special Thick Plate Technology. J. Univ. Sci. Technol. B.

[13] Zu G., Li H., Li B. (2007). Effect of High-frequency Current Heating Online Process on Microstructures and Mechanical Property of Stainless Steel/Carbon Steel Cladding Strip. Acta Metall. Sin..

[14] (2008). Clad Steel Plates- Mechanical and Technological Test.

[15] Peng J., Luo S., Yuan M. (2007). Research on Oxidation Behavior of 304 Austenitic Stainless Steel at High Temperature. Baosteel Technol..

[16] Tang C.H., Hou L.L. (2008). Reason Analysis about Intergranular Corrosion on Stainless Steel Side of 316L-16MnR Cladding Plate. Phys. Test. Chem. Anal..

[17] He X.S., Li P.C., Zhao H. (2015). Analysis on Effect of Heat Treatment Processes on Inter-crystal Line Corrosion of Stainless Steel Composite Plate. Hot Work Tech..

[18] Liu Z.C., Wang H.Y., Ren H.P. (2009). Surface Convexity of Supercooled Austenite Transformation Products. C Stereol. Image Anal..

[19] Li T.F. (2003). High Temperature Oxidation and Hot Corrosion of Metals (Corrosion and Protection).

[20] Ellingham H.J. (1944). Reducibility of oxides and sulfides in metallurgical processes. Soc. Chem. Ind..

[21] Liu Z.C., Ji Y.P., Wang H.Y., Ren H.P. (2011). The Fourth Commentary on Shear Mechanism of Martensite Phase Transformation. Heat Treat. Met..

[22] Lin X.Q., Liu Z.C. (2009). Martensite Floating and Forming Mechanism of Fe-1.5Ni-0.6C Alloy. J. Inner Mong. Univ. Sci. Technol..

[23] Xin J.P. (2018). The Experiment Analysis on Composition of Floating Substance in Casting Process of Aluminum Deoxidized steel. Heavy Cast. Forg..

[24] Nomura M., Hashimoto I., Kozuma S. (2006). Effects of Surface Oxides on the Phosphatability of the High Strength Cold Rolled Steels (Surface Treatment and Corrosion). Tetsu-to-Hagane.

[25] Wild R.K. (1977). High Temperature Oxidation of Austenitic Stainless Steel in Low Oxygen Pressure. Corros. Sci..

[26] Jian P., Jian L., Bing H. (2006). Oxidation Kinetics and Phase Evolution of a Fe-16Cr alloy in Simulated SOFC Cathode Atmosphere. J. Power Source.

[27] Kocsis V., Bordacs S., Varjas D. (2013). Magnetoelasticity in ACr(2)O(4) Spinel Oxides (A = Mn, Fe, Co, Ni and Cu). Phys. Rev. B.

[28] Adams D.P., Hodges V.C., Hirschfeld D.A. (2013). Nanosecond Pulsed Laser Irradiation of Stainless Steel 304L: Oxide Growth and Effects on Underlying Metal. Surf. Coat. Technol..

